# Supplementary material for: A Global Screen for Assembly State Changes of the Mitotic Proteome by SEC-SWATH-MS
Source: Cell Syst. 2020 Feb 26;10(2):133–155.e6. doi: 10.1016/j.cels.2020.01.001 (PMC7042714; doi:10.1016/j.cels.2020.01.001)

Q7KZ85 | SPT6H_HUMAN | SUPT6H KIAA0162 SPT6H
Monomer MW [kDa]: 199.073 Monomer expected elution fraction: 34

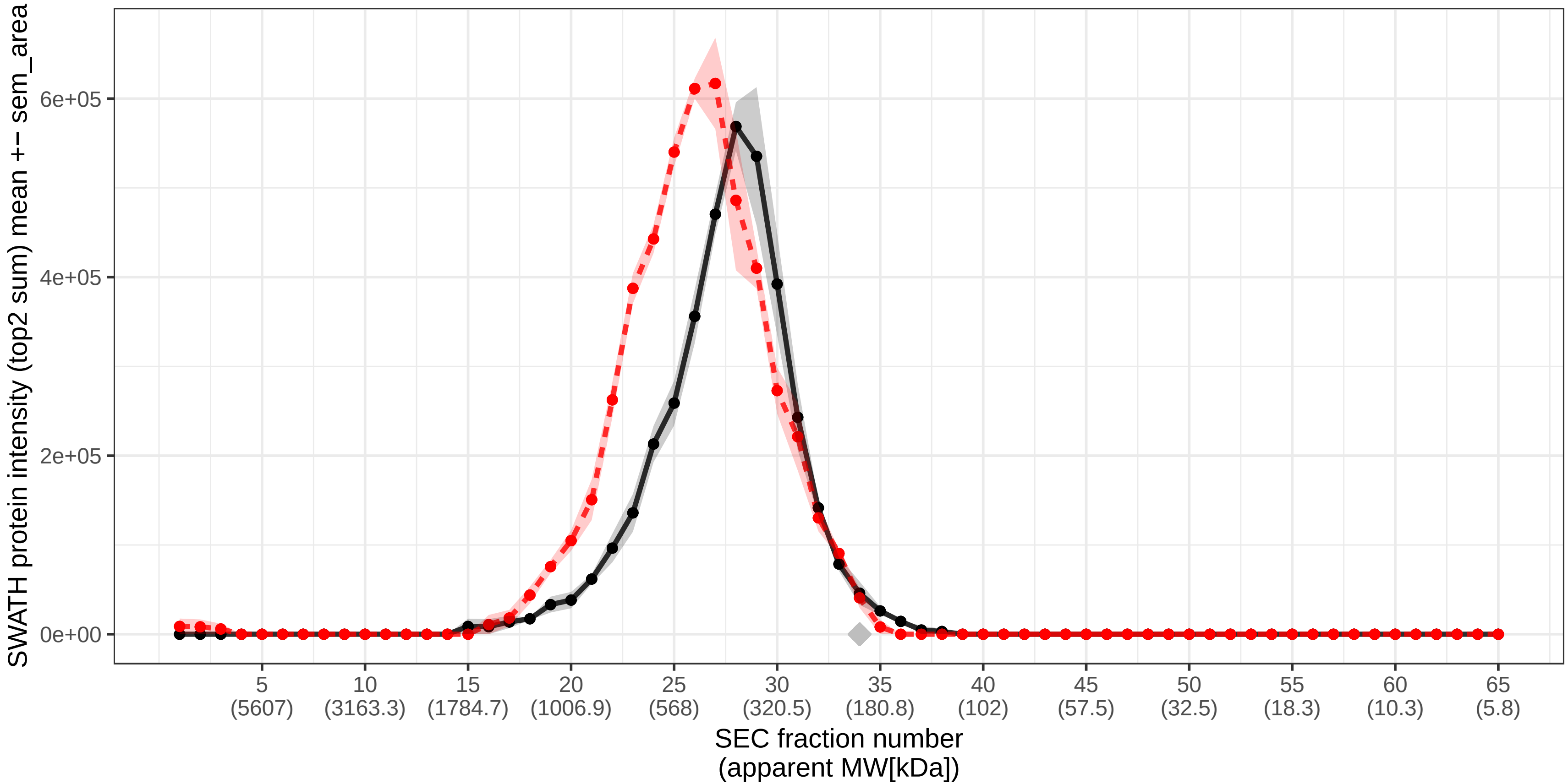

Supplement: Data S1. SEC-SWATH-MS Protein Chromatograms, Related to Figure 1 [file mmc6.zip › SECchrom_Q7KZ85_SPT6H_HUMAN_SUPT6H_KIAA0162_SPT6H.pdf]
